# Evolutionary and Spatiotemporal Dynamics of Foot‐and‐Mouth Disease Virus in Ethiopia (1957–2025): A Molecular Perspective on Serotype Persistence and Emergence

**DOI:** 10.1155/tbed/6349383

**Published:** 2026-09-26

**Authors:** Daniel Gizaw, Fentahun Wondmnew, Fasil Aklilu, Getnet Abie Mokonnen, Nina Henning, Berhanu Admasu, Badi Maulidi, Wudu Temesgen, Jef. Hammond

**Affiliations:** ^1^ Animal Health Institute (AHI), Sebeta, Ethiopia, ahi.org; ^2^ Global Alliance for Livestock Veterinary Medicines (GALVmed), Edinburgh EH26 0PZ, Scotland, UK; ^3^ Infectious Diseases and Global Health, Africa Regional Office, Cummings School of Veterinary Medicine/ Tufts University, Addis Ababa, Ethiopia; ^4^ International Livestock Research Institute (ILRI), Addis Ababa, Ethiopia, ilri.org; ^5^ University of Gondar, Gondar, Ethiopia, uog.edu.et

**Keywords:** Ethiopia, foot-and-mouth disease, molecular epidemiology, serotype dynamics, topotypes, transboundary animal diseases

## Abstract

Foot‐and‐mouth disease (FMD) represents a significant global economic burden and remains the primary constraint on Ethiopia’s livestock economy and international trade. This desk review synthesized 60 years of data (1957–2025) using a structured, multidatabase search across four domains: disease, genotype/lineage, geographic spread, and temporal context. Data were stratified into three periods: Period I (1957–1980), Period II (1981–2000), and Period III (2001–2025). Results indicate that Period I saw the identification of serotypes O, A, and C, though serotype C has not been detected after 1983. Period II was marked by the continued circulation of serotypes O and A, and the first emergence of Serotype SAT 2 near the Kenya–Ethiopia border, which temporarily disappeared until its major re‐emergence in 2007. Period III is characterized by a significant intensification and modernization of FMD virus (FMDV) outbreak investigation that provided more granular data on viral activity in the country. The period saw four major endemic serotypes O, A, SAT 2, and SAT 1, SAT one being confirmed for the first time during this period, circulate simultaneously and covering the entire country. Molecular analyses revealed a heterogeneous landscape: serotype O is dominated the serotype distribution in the country by its O/EA‐3 and O/EA‐4 topotypes, while serotype A involving complex lineages (Africa G‐I, G‐IV, and G‐VII) follows in serotype O and precedes serotype SAT 2 in distribution. Notably, serotype SAT 2 has historically comprised four topotypes (IV, VII, XIII, and XIV), with the recent dominance of the re‐emergent SAT 2/XIV topotype. The global biosecurity threat posed by FMDV SAT 2 topotype XIV stems from its unprecedented transcontinental dissemination into immunologically naive regions, rather than inherent lethality. This expansion highlights the complex evolutionary dynamics of the virus and the vulnerability of current surveillance system. The global dissemination of emerging lineages, such as SAT 2 topotype XIV, underscores the complex evolutionary dynamics and environmental plasticity inherent across all FMDV serotypes. The rapid shift of these viruses into nonendemic territories highlights critical vulnerabilities in existing biosecurity. Ultimately, these dynamics necessitate a shift toward adaptive, multiserotype surveillance and integrated control frameworks to address the continuous antigenic evolution and geographical expansion of the virus. Advancing Ethiopia along the progressive control pathway (PCP‐FMD) is critically contingent upon evaluating the protective efficacy of current trivalent vaccines against rapidly evolving field strains. Immediate intervention is required to address diverged genotypes that jeopardize both national herd health and international market access.

## 1. Introduction

Foot‐and‐mouth disease (FMD) is a highly contagious viral infection that affects a broad spectrum of cloven‐hoofed animals, including major domestic species (cattle, swine, sheep, and goats). Despite low mortality, FMD is a disease of significant global concern, imposing a massive annual economic loss estimated between US$ 6.5 and US$ 21 billion [1]. This colossal global economic burden is primarily driven by reduced animal productivity, high surveillance and prevention costs, and strict restrictions on international trade [1, 2].

The FMD virus (FMDV) exists in seven immunologically distinct serotypes [3], which are further organized into seven regional pools representing independent ecosystems where similar viral lineages circulate and interact [4, 5]. The geographical variation in FMDV is nonuniform, differing substantially across endemic and nonendemic regions [6]. To facilitate a standardized understanding of the classification used in this review, the following terminology is applied:

### 1.1. Serotype

This refers to the seven immunologically distinct FMDV types (O, A, C, SAT 1, SAT 2, SAT 3, and Asia 1). Infection with one serotype does not confer cross‐protection against the others.

### 1.2. Topotype

A geographical classification based on phylogenetic analysis of the VP1 coding sequence is typically defined by >15% nucleotide divergence from other topotypes within the same serotype.

### 1.3. Genotype/Lineage

Hierarchical subclassifications within a topotype represent closely related viral clusters that often share a common evolutionary origin and geographic or temporal association.

### 1.4. FMDV Pools

A global classification system was adopted by the World Organization for Animal Health (WOAH) and the Food and Agriculture Organization (FAO) to categorize FMDV circulation into seven distinct ecosystems. These pools are often defined by ecological or geographical barriers, providing a framework to guide vaccine matching and regional control strategies.

The FMD challenge is particularly intricate in Africa, which documents six of the seven known FMDV serotypes (O, A, C, SAT 1, SAT 2, and SAT 3) [7] and where molecular analyses show continuous viral dispersion [8]. The epidemiology is complex due to transmission between domestic animals and wildlife, notably the African buffalo (*Syncerus caffer*), which is the natural maintenance host for SAT viruses [9, 10], alongside an independent domestic cycle. In comparison, Asia primarily manages serotypes O, A, and Asia‐1 [5] However, the global FMDV risk landscape has shifted significantly since 2025 due to the rapid, transboundary expansion of the SAT serotype beyond its traditional sub‐Saharan African range. Following its official detection in Iraq in early 2025 [11, 12], SAT‐1 disseminated through the Middle East, West Asia, and the Caucasus, with confirmed detections in parts of Europe and East Asia by early 2026 [13, 14]. This geographic spread highlights the role of livestock movement in long‐distance dissemination, underscoring the extreme vulnerability of immunologically naïve populations to novel topotypes and the critical gaps in global surveillance and vaccine cross‐protection.

The FMDV serotype O is widespread across Africa, with various topotypes characterizing regional prevalence. The O/EA‐4 topotype comprises the viruses found in Ethiopia [15, 16], while other variants of serotype O, specifically the EA‐1 and EA‐2 topotypes, were characterized in Eastern African countries, including Uganda [17] and Kenya [18]. Serotype O is also broadly reported across East, West, Central, and North Africa [19–21].

Serotype A viruses in Africa show complex circulation, with genotype A (Africa G‐I) identified in Uganda [17] and genotype IV reported in Egypt [22] and Nigeria [19]. Furthermore, genotypes A‐IV, V, and VII have been exchanged among North, Central, and West African countries.

There is a frequent co‐occurrence of serotypes O, A, and SAT 2 within specific FMDV pools [21, 23, 24]. While the SAT serotypes (SAT 1, SAT 2, and SAT 3) were historically known for localized spread [25], their incursion outside Africa, particularly into the Middle East, is now frequent. SAT 2 is widely reported across Africa, including Nigeria, Libya, Cameroon, Ghana, Uganda, and Ethiopia [16, 17, 19, 26], with dissimilar topotypes circulating in East, South, Central, and West Africa [27, 28].

FMDV was first officially reported in Ethiopia in 1957 with the identification of serotypes O, A, and C [29]. While clinical cases were likely present in the country prior to this date, 1957 marks the beginning of documented surveillance and serves as the baseline for this analysis. Following these initial reports, Ethiopia has experienced decades of endemic circulation, characterized by the persistent presence of multiple circulating serotypes [16, 30].

Currently, the disease is maintained by the sustained circulation of three major serotypes: O, A, and SAT 2; serotype C has not been reported in Ethiopia since 1983, which is consistent with its apparent global disappearance following the last confirmed outbreaks in Kenya and Brazil in 2004 [31].Despite this long‐standing endemic status, there remains a significant research gap; most FMD studies in the country rely on serological findings, with a paucity of robust phylogenetic analysis and molecular characterization of the circulating viral strains [15, 16, 29, 30].

In Ethiopia, serotype O is consistently reported as the most prevalent serotype during outbreaks, followed by serotype A and SAT 2 [6, 15, 16]. Serotype A is widely circulating alongside serotype O [6, 15, 16, 32–34]. Serotype A viruses in Ethiopia are phylogenetically classified within the Africa topotype, specifically clustering into genotypes G‐I, G‐IV, and G‐VII. While G‐IV and G‐VII are more prevalent, genotype G‐I represents a rarer occurrence, documented only once during a 2018 outbreak [16]. Conversely, Serotype SAT 1 has been characterized only once by sequencing in 2007, grouping to a distinct topotype IX [15], despite multiple serological detections since then [6, 15, 16, 32–34].

The complex distribution of these viral topotypes is closely linked to livestock movement, ecosystem diversity, and pastoralist adaptations to seasonal trends [6, 35], with cross‐border animal movement between Ethiopia and its neighbors (Sudan, Kenya, Somalia, and Djibouti) serving as a significant driver for FMD circulation.

FMD has a high impact on livestock in Ethiopia, causing morbidity and economic loss differently across production systems [36–38]. The core objective of this review is to synthesize and critically evaluate the historical and current epidemiology of FFMDV and surveillance efforts in Ethiopia, focusing specifically on circulating serotypes from 1957 to 2025. The resulting findings will then be used to establish evidence‐based, risk‐stratified strategies that inform the technical roadmap for Ethiopia’s accelerated progression along the OIE/FAO PCP‐FMD.

## 2. Materials and Methods

### 2.1. Review Design

This review employed a systematic literature review design to comprehensively collect, assess, and synthesize existing evidence on FMDV serotypes circulating in Ethiopia between 1957 and 2025. This methodology was specifically chosen to consolidate both historical and contemporary data regarding FMDV distribution, diagnostic evolution, and epidemiological trends. The study utilized a decadal analytical framework to evaluate temporal trends in FMDV distribution over 10‐year increments, highlighting shifts in dominant serotypes, diagnostic technologies, and geographic distribution over time. The entire review was strictly conducted following established guidelines for systematic evidence synthesis and secondary data analysis, thereby ensuring comprehensiveness, transparency, and reproducibility [39].

### 2.2. Data Sources

Information for this systematic review was obtained from both online and offline repositories to ensure comprehensive coverage of the relevant literature and reports. Digital databases consulted for the peer‐reviewed literature included PubMed, Google Scholar, Directory of Open Access Journals (DOAJ), and CABI Abstracts. Gray and institutional literatures were retrieved from Ethiopian organizations, specifically the Animal Health Institute (AHI), the National Veterinary Institute (NVI), and the Ministry of Agriculture (MoA). In addition, international databases and reference laboratories, including the World Reference Laboratory for FMD (WRLFMD) at the Pirbright Institute, were systematically accessed to incorporate global data and nondigitized reports.

### 2.3. Search Strategy

A comprehensive multidatabase search was conducted using structured Boolean operators to combine four major conceptual domains: disease, lineage/genotype or subtyping, circulation or geographic spread, and temporal dynamics. The search was last updated on October 17, 2025. The core PubMed search string used was:(“Foot and Mouth Disease” OR FMD OR FMDV OR Aphthovirus) AND (serotype OR serotypes OR lineage OR lineages OR topotype OR genotype OR “molecular epidemiology” OR “virus isolation”) AND (circulation OR outbreak OR “risk factors” OR epidemiology OR “spatial distribution” OR spatiotemporal) AND (Ethiopia OR Ethiopian). Equivalent search strings were adapted for other databases, including CABI Abstracts, Google Scholar, and DOAJ, to ensure syntax compatibility with each platform. The initial search yielded 326 records: 49 from PubMed, 163 from Google Scholar, 73 from CABI Abstracts, and 41 from DOAJ.

### 2.4. Data Extraction and Synthesis

A standardized data extraction template was developed in Microsoft Excel to ensure consistency and minimize bias. Extracted variables were designed to capture the necessary context, including the year or decade of the outbreak/study, precise geographic location, affected animal species, FMDV serotype(s) and/or topotype(s), and the specific diagnostic methods employed (e.g., virus isolation, ELISA, polymerase chain reaction [PCR], or VP1 sequencing). To examine historical patterns, the extracted data were systematically stratified into three temporal periods: Period I (1957–1980), Period II (1981–2000), and Period III (2001–2025). Quantitative data were summarized using descriptive statistics and visualized via tables, bar graphs, and line charts to illustrate temporal and spatial variations. A narrative synthesis was then employed to interpret these patterns, specifically highlighting the emergence, persistence, or decline of specific FMDV serotypes and topotypes over time.

For the purpose of this review, the primary unit of analysis is a “documented diagnostic event,” defined as a single confirmed laboratory result for FMDV. Given the seven‐decade scope of this study, the definition of an “outbreak” varies significantly across the literature, ranging from a laboratory‐confirmed case in a single animal to clusters of infected herds within a district. Consequently, each reported diagnostic event was treated as an independent record. It is important to emphasize that these records represent a longitudinal snapshot of viral circulation rather than a systematic measure of incidence or prevalence. The inherent variation in sampling intensity—reflecting a shift from passive clinical reporting in early decades to targeted molecular surveillance in more recent years is a critical consideration when interpreting the frequency counts presented in Tables 1–3.

**Table 1:** Foot‐and‐mouth disease serotype distribution: 1957–1980 (Period I).

No.	Reference	Year of outbreak	Location	FMD virus serotype				
				O	A	C	SAT 1	SAT 2
1.	[29]	1957	Addis Ababa, Benchi Maji, Surma	18	—	1	—	—
2.	[29]	1969	Sidama	8	—	1	—	—
3.	[29]	1971	North Shoa, Benchi Maji, Surma	36	—	23	—	—
4.	[15]	1974	Addis Ababa	0	1	—	—	—
5.	[30, 40]	1979	Adama	2	—	—	—	—
Total				64	1	25	1	1

**Table 2:** Foot‐and‐mouth disease serotype distribution: 1980–2000 (Period II).

No.	Reference	Year of outbreak	Location	FMD virus serotype				
				O	A	C	SAT 1	SAT 2
1.	[15]	1981	Gefersa	—	2	—	—	—
2.	[15]	1982	Adama, Debre Berhan	10	8	—	—	—
3.	[15, 40]	1983	Debre Berhan, Surma	5	—	3	—	—
4.	[15]	1984	Benchi Maji, Addis Ababa	—	7	—	—	—
5.	[15]	1985	Bahir Dar, Borana	—	7	—	—	—
6.	[15]	1986	Benchi Maji, Negelle Borana, Debre zezit	2	2	—	—	—
7.	[15]	1987	Bahir Dar, Wolayita sodo	6	—	—	—	—
8.	[15]	1988	Debre Zeit, Adama	3	—	—	—	—
9.	[15]	1989	Wolaita, Yabelo	3	—	—	—	2
10.	[15, 30, 40]	1990	Adama, Modjo, Borana, Sidama	18	—	—	—	4
11.	[15, 30]	1991	Yabelo, Debrebrehan, Addis Ababa	2	—	—	—	6
12.	[15]	1992	Modgjo, Meki, Sebeta	13	1	—	—	—
13.	[15, 40]	1993	Debre Brehan, Woyilat Sodo	5	—	—	—	—
14.	[15, 40]	1994	Meki, Adama	13	12	—	—	—
15.	[15, 40]	1995	Wolaita Sodo, Fiche	7	—	—	—	—
16.	[15, 40]	1996	Adama, Western Tigray, Debremakose	2	1	—	—	—
17.	[15]	1998	Fiche, Arbaminchi	10	—	—	—	—
18.	[15]	1999	Debre Markos	16	—	—	—	—
19.	[15]	2000	Arbamichi, Gefersa Dire Dawa	3	3	—	—	—
Total				118	43	3	—	12

**Table 3:** Foot‐and‐mouth disease serotype distribution: from 2001 to 2025 (Period III).

No.	Reference	Year of outbreak	Location	FMD virus serotype				
				O	A	C	SAT 1	SAT 2
1.	[15, 30, 40]	2001	Adama, Hadiya, Konso	15	3	—	—	—
2.	[15]	2002	Addis Ababa, Gefersa	—	1	—	—	—
3.	[15]	2003	Mizan Tefere, Borana	3	—	—	—	—
4.	[15]	2004	Guba Lafto, Negela Borana	26	—	—	—	—
5.	[15]	2005	Sebeta, Robe Arsi	20	—	—	—	—
6.	[15]	2006	Adama Yabelo	11	—	—	—	—
7.	[15, 41]	2007	Ankasha, Awi, Surma Sheka, Western Tigray, Adaba, Assosa, Benci Maji, Fiche	22	4	—	9	1
8.	[16, 32, 34]	2008	Sekela, Adet, Dire Dawa, Borana, Sirka, Gebella	46	15	—	—	—
9.	[16, 32, 34]	2009	East Hararghe, Adama, Meki, Deber berhan	54	14	—	—	37
10.	[16, 32, 34, 42, 43]	2010	Debreberhan Wolyata Sodo, Kucha, Addis Ababa, Arbaminich	16	—	—	—	31
11.	[16, 32, 34]	2011	Benchi Maji, Mekele Borana	85	—	—	—	—
12.	[16]	2012	Woldeya,	13	—	—	—	—
13.	[16]	2013	Bishoftu,	49	—	—	—	3
14.	[16]	2014	Dinisho, East Wollega	1	—	—	—	—
15.	[16, 33]	2015	Fafan Zone, Addis Ababa Guna, Adama, Sidama	10	9	—	—	21
16.	[16, 33, 44]	2016	Debre Brehan, Bita Genet	33	—	—	—	—
17.	[16, 45]	2017	Kellom Wollega, Este, Makisenyit, Mekele Bahir Dar	35	3	—	—	1
18.	[16, 46, 47]	2018	Southern Tigray, Gaweta, Sendafa, Meki, Humera	43	53	—	—	—
19.	[16, 47–50]	2019	Kombolcha, Eastern Tigray, Bishoftu Humera, Horuguduru, Aba’ala	38	24	—	—	16
20.	[16, 51]	2020	Sodo, Guragae, Sheger citify, Silte Horuguduru, Jima	23	18	—	—	—
21.	[6, 16, 52, 53]	2021	Walayita, Addis Ababa Wolkite, Debre Berhan, Jima	47	22	—	—	40
22.	[6, 54]	2022	Assos, Halaba, Sseden Sodo, Wolayita Sodo	2	—	—	—	52
23.	[6]	2023	Holaba, Arsi, Horuguduru	14	14	—	—	14
24.	AHI (unpublished data)	2024	Huruguduru, Sebeta, Horuguduru	54	5	—	—	1
25.	AHI (unpublished data)	2025	Afar, Arsi Debrhebrehan,	49	3	—	—	—
Total				709	188	—	9	217

*Note:* For the three tables, the number of isolates does not indicate the severity or the extent of the outbreak over time. It only represents the number of positive samples.

## 3. Result and Discussion

### 3.1. Screening, Inclusion, and Exclusion Criteria

**Figure 1:**
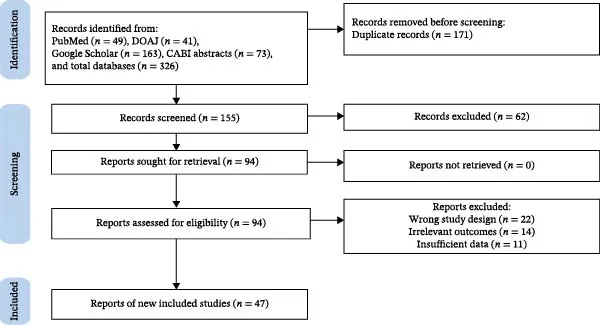
Schematic representation of the literature selection procedure adopted for the systematic review of FMD prevalence in Ethiopia.

The selection of documents was performed in two stages to ensure their relevance and quality. The process began with a preliminary screening based on titles and abstracts to exclude irrelevant studies, such as those conducted outside Ethiopia or those lacking FMDV serotype identification data (including serological studies). This was followed by a full‐text evaluation of potentially eligible studies against specific inclusion criteria. The included studies were strictly those reporting FMDV occurrence within Ethiopia between 1957 and 2025 and providing laboratory‐confirmed serotype identification (specifically O, A, SAT 1, SAT 2, and C), along with sufficient metadata (location, host species, and diagnostic methods). Additionally, manual snowball searching of reference lists from key studies and institutional reports was performed to capture historical data, particularly for pre‐1980 outbreaks. Following the full‐text evaluation and application of all criteria, 47 studies were retained for the final synthesis and analysis (Figure 1).

Publications without clear serotype data, unclear diagnostic methodology, or restricted to other countries without cross‐border relevance were excluded. Opinion papers, editorials, and duplicate versions of the same articles were also removed, and the most complete sources were retained for data extraction.

### 3.2. Temporal Distribution of FMDV Serotypes in Ethiopia (1957–2025)

**Figure 2:**
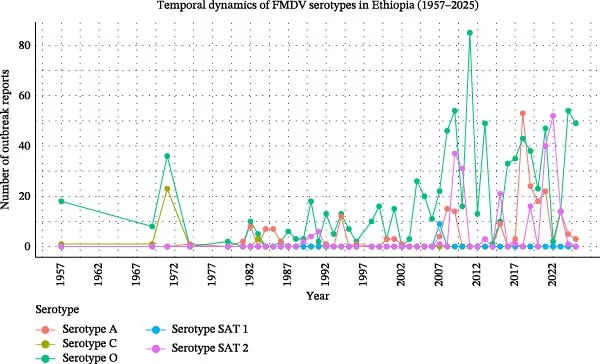
Temporal dynamics and serotype diversity of FMDV reports in Ethiopia, 1957–2025.

This comprehensive review, covering the dynamics of FMDV outbreak investigations in Ethiopia, reveals a distinct temporal shift in serotype prevalence (Figure 2), the initial period (1957–1980) established the foundational understanding, with the identification of serotypes O, A, and C in regions like Addis Ababa, Benchi Maji, and Surma (Table 1). Serotype C, with peak isolates reported in 1971, represents a critical early finding. Its subsequent, complete disappearance after 1983 marks a significant epidemiological event [15, 29].

This trend provides a clear transition into the second period (1981–2000), Period II, which is characterized by the conspicuous absence of serotype C and the confirmed, continued circulation of serotypes O and A [15, 40], thereby defining a new endemic baseline for FMDV threats in the country.

The second period is further distinguished by the initial emergence of the SAT 2 serotype (Table 2), marking an expansion of the FMDV serotype pool in Ethiopia. The review clearly indicates an early expansion pattern, originating near the Ethiopian–Kenyan border in Yabelo, followed by a sustained geographical push toward central Ethiopia, with SAT 2 subsequently detected in locations like Adama, Modjo, Sidama, Addis Ababa, and Debre Birhan throughout 1990 and 1991 [15, 30]. This suggests a significant, albeit temporary, incursion, and spread route. However, despite the continued, consistent reporting of serotype O throughout this same period, the presence of SAT 2 proved to be ephemeral; it was not detected again until its reoccurrence during major outbreaks starting in 2007 [15, 16].

The third and current period of the review (2001–2025) is unequivocally characterized by a significant intensification and modernization of FMDV outbreak investigation, marked by a substantial increase in the research community’s engagement. This era saw the systematic adoption of sophisticated diagnostic and characterization techniques, including antigen detection, virus isolation, PCR, and phylogenetic analysis, moving the understanding of FMDV from mere detection to molecular epidemiology. Crucially, throughout this period of enhanced surveillance, a major epidemiological finding confirmed across all reported studies is the unwavering and persistent dominance of FMDV serotype O [15, 16, 32, 36]. Data spanning diverse regions consistently show that serotype O has maintained its position as the principal circulating strain, underscoring its role as the primary endemic threat in Ethiopia and emphasizing the need for focused control strategies.

The third period highlights a significant shift in the FMDV epidemiological landscape with the novel introduction and characterization of serotype SAT 1 in 2007 (Table 3). This emergence was first recorded in the southern regions of Benchi Maji and Surma [15, 16, 32, 36, 41]. Critically, this finding confirms that all four major endemic serotypes O, A, SAT 2, and the newly established SAT 1 were simultaneously circulating across various parts of the country. The identification of SAT 1 solidified a more complex and diverse endemic profile for FMDV in Ethiopia, presenting a substantial challenge to national control and vaccination strategies. It is noteworthy that the most recent data (2024–2025; Table 3) were generated at the AHI using ISO 17025‐accredited molecular diagnostic protocols and Antigen detection ELlSA (Ag ELISA), ensuring rigorous quality control and technical validation consistent with international standards.

**Figure 3:**
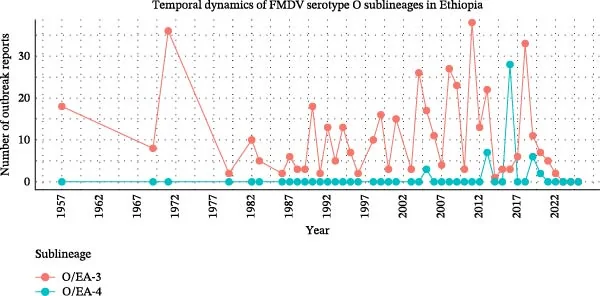
Temporal dynamics of foot‐and‐mouth disease virus serotype O lineages, 1957–2025.

The FMDV outbreak investigation during Period III provides a granular view of viral activity, particularly regarding the temporal trend of isolates detected and characterized after 2010 (Figure 3). This phase was marked by a peak in serotype O isolation in 2011, with an exceptional 85 isolates recorded within that single year. Furthermore, spatiotemporal analyses corroborate this high activity, identifying 2011 as the year with the highest recorded number of FMDV outbreaks across the country [55, 56].

Despite this dramatic peak, the subsequent decade saw serotype O only briefly and seldom overtaken by serotypes A and SAT 2; the long‐term trend confirms that serotype O maintained its status, remaining the consistently dominant serotype over the entire period [16, 32, 55]. This pattern highlights serotype O’s endemic persistence, punctuated by occasional sharp epidemic increases like the one observed in 2011, while demonstrating the continuous, yet subsidiary, threat posed by serotypes A and SAT 2.

Further analysis of the temporal trends in Period III reveals significant secondary peaks for the non‐dominant serotypes, A and SAT 2, demonstrating their ongoing epidemic potential. Serotype A showed its highest isolation event in 2018, with a substantial total of 53 isolates collected within that year. This peak activity was geographically widespread, originating from diverse locations including Southern Tigray, Gaweta, Sendafa, Meki, and Humera [16, 57]. Similarly, serotype SAT 2 isolation peaked in the latter part of this period, with a peak of 52 isolates reported in 2022 (Table 3). This surge was associated with outbreaks in various distinct regions, including Assosa, Halaba, Seden, Sodo, and Wolayita Sodo [6, 54]. These findings collectively underscore the fact that while serotype O maintains overall long‐term dominance, serotypes A and SAT 2 possess the capacity to drive intense, geographically distributed epidemic waves in specific years, demanding continuous, serotype‐specific surveillance and preparedness.

A crucial finding of this review concerns the status of serotypes C and SAT 1, both of which demonstrate either complete disappearance or highly ephemeral presence. Serotype C, first described in 1957 [29], has been entirely absent from isolation records since 1983, with its last detection occurring in Bench Maji and Surma [15, 30]. The extended absence of this serotype aligns with a global cessation of detection since 2004. This suggests a comprehensive clearance from the worldwide FMDV endemic pool, indicating that the lineage is no longer circulating internationally [31]. The status of serotype SAT 1 is defined by its brief, transient presence. First isolated and identified in 2007 in the Bench Maji, Yabelo, and Surma regions [15, 41], no subsequent isolates have been genetically confirmed since its initial emergence. Although limited serological evidence has occasionally suggested low‐level circulation [16, 33], recent comprehensive surveillance raises critical doubts regarding its endemic status.

Specifically, Gizaw et al. [6] in their study focusing on FMDV serotypes and genetic diversity (2019–2023) investigated the accuracy of detection methods. The study reported that while the antigen‐ELISA (Ag‐ELISA) test initially yielded positive results for SAT 1 in seven samples, subsequent molecular sequencing failed to corroborate any outbreaks caused by the serotype. Consequently, the authors proposed that the Ag‐ELISA results were likely false positives, reinforcing the conclusion that SAT 1 is not a consistently circulating strain in the country.

This comprehensive review synthesizes data from the first recorded detection in 1957 through the most recent reports in 2025, unequivocally confirming the long‐term, endemic status of FMDV in Ethiopia. The detailed serotype distribution presented in Tables 1–3 documents a complex, multi‐serotype epidemiology, a defining feature of FMDV circulation in the country. While surveillance efforts have varied over the past six decades and recorded outbreak data may not capture the full picture of disease distribution, the tabulated locations serve as critical data points, identifying specific regions where the circulation of particular FMDV serotypes was confirmed. This structured compilation of historical and current reports is vital for epidemiological risk assessment, demonstrating the persistent, diverse viral threat that underpins the necessity for continuous, targeted disease control strategies.

Overall, this comprehensive review synthesizes data from the first recorded detection in 1957 through to the most recent reports in 2025 and unequivocally confirms the long‐term, endemic status of FMDV in Ethiopia. Crucially, the detailed serotype distribution presented in Tables 1–3 documents a complex, multiserotype epidemiology, which is a defining feature of FMDV circulation in the country. While surveillance efforts have varied considerably over the past six decades and the recorded outbreak data might not present the complete picture of the disease distribution, the tabulated locations serve as critical data points, identifying specific regions where the circulation of a particular FMDV serotype was confirmed. This structured compilation of historical and current serotype reports is vital for epidemiological risk assessment, demonstrating the persistent, diverse viral threat that underpins the necessity for continuous, targeted disease control strategies.

Data for 2024–2025 labeled as “AHI unpublished data” were generated from laboratory‐confirmed FMD outbreak investigations conducted by the AHI, Ethiopia, using field samples collected from affected areas including Horro Guduru, Sebeta, Afar, Arsi, and Debre Berhan. Serotype identification was performed using routine diagnostic procedures, including antigen ELISA and RT‐PCR. In 2024, 54 serotype O isolates, five serotype A isolates, and one SAT 2 isolate were detected, while in 2025, 49 serotype O isolates and three serotype A isolates were identified. These data were included to provide an updated epidemiological context but have not yet undergone formal, peer‐reviewed publication.

### 3.3. Temporal Trend of FMDV Serotype O Lineages in Ethiopia (1957–2025)

The molecular characterization of FMDV serotype O isolates in Ethiopia reveals a circulation pattern dominated by two distinct regional topotypes: East Africa 3 (EA‐3) and East Africa 4 (EA‐4) [6, 15, 16]. The geographical distribution and temporal history of these topotypes demonstrate complex epidemiological dynamics within the country.

Topotype O/EA‐3 represents the dominant lineage circulating in Ethiopia [6, 15, 16]. Its establishment is historic, dating back to the first documented outbreak investigation in 1957, which was the initial identification of the EA‐3 lineage in the country [29]. This lineage has since revealed endemic circulation across nearly all regions of Ethiopia. The broad prevalence of O/EA‐3 is significant because the current FMD vaccine strain used locally is based on this specific lineage [6, 15, 16], suggesting that it provides the most appropriate coverage against the majority of circulating serotype O infections. In contrast, the O/EA‐4 topotype exhibits a more restricted, localized distribution, primarily reported in the central and southwest Oromia, and the Southern Nations, Nationalities, and Peoples (SNNP) regions [6, 15, 16]. The detection of the EA‐4 lineage is relatively recent, first documented in 2005, with subsequent reports in 2013, 2016, and 2020 [6, 15, 16, 33, 44]. A critical finding in the molecular epidemiology of serotype O is the concurrent circulation of both O/EA‐3 and O/EA‐4 topotypes in most documented outbreaks where EA‐4 was reported (i.e., 2005, 2013, and 2020), with the year 2016 being the only exception. This cocirculation, often within the same locations, highlights the potential for antigenic competition or synergistic disease maintenance. The restricted geographical range of O/EA‐4, compared to the widespread distribution of O/EA‐3, suggests differing pathways of introduction or lower adaptive fitness/transmission efficiency, although its persistent presence warrants continued molecular monitoring to assess the risk of a wider geographic spread and potential vaccine mismatch. The temporal trends are shown in (Figure 3).

### 3.4. Temporal Trend of FMDV Serotype A Lineages in Ethiopia (1957–2025)

**Figure 4:**
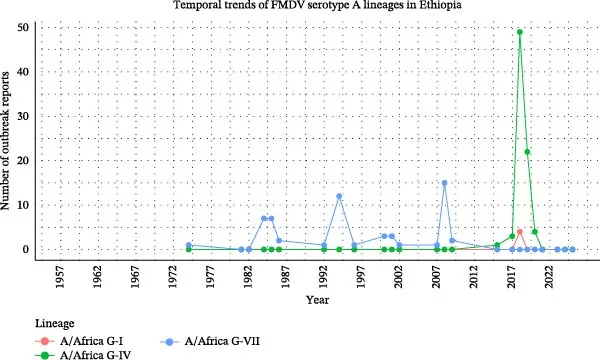
Temporal dynamics of foot‐and‐mouth disease virus serotype A lineages, 1957‐2025.

Serotype A of FMDV represents a major component of the endemic circulation in Ethiopia, initially isolated by Martel (1974) and subsequently characterized in detail by Ayelet et al. [15]. A key epidemiological feature of this serotype is its widespread and cyclical recurrence, often circulating concurrently with serotype O [15, 16, 32]. Serotype A’s temporal trends across the period of 1957–2025 reveal three distinct phases of activity (Figure 4). The first two decades (1957–1980) were marked by dormancy; this was followed by a phase of emergence and low‐level activity from 1980 to 2000, which included minor peaking around 1985–1990. The most significant and dynamic phase commenced around 2005 and was characterized by high variability and increased recurrence. This period led to the highest recorded frequency for serotype A, culminating in a major peak of approximately 53 reports around 2019, a surge likely reflective of intensified FMD outbreak investigation efforts during this time [15, 16, 32, 58]. However, more recent, unpublished data indicates a sharp decline in serotype A reports between 2022 and 2025.

The recent epidemiological pattern of serotype A underscores the highly dynamic nature of FMD in the country, with its annual prevalence fluctuating markedly. While serotype A was the most prevalent serotype reported in 2019 [6], its profile shifted in 2020 as serotype O became more dominant, preceding the expansion of serotype SAT 2 from 2021 to 2023 [6]. This serotype flux is also reflected in localized studies that reported increased FMD outbreaks overall in 2019 [59–61]. Furthermore, overall surveillance data spanning 2008–2019 confirms serotype A’s position as the second most prevalent serotype (*n* = 51), following serotype O (*n* = 175) and preceding serotype SAT 2 (*n* = 33) [16], highlighting its persistent role as a major etiological agent.

Molecular characterization via VP1 sequencing classifies FMDV serotype A into multiple genotypes within the Africa topotypes, including A/Africa G‐I, G‐IV, and G‐VII [15, 16, 57, 58], of these, the A/Africa G‐IV lineage is considered endemic and well‐established in Ethiopia [6, 15, 16, 57, 58]. This endemic status is supported by the monophyletic clustering of A/AFRICA/G‐IV sequences from 2018–2022, a clade composed exclusively of Ethiopian isolates [6], suggesting a sustained, indigenous transmission cycle. The A/Africa G‐VII lineage has also been consistently characterized, while A/Africa G‐I appears to be a sporadic identification, reported only from a single outbreak in 2018 [6]. The dominance of G‐IV, with other A genotypes largely undetected since 2010, affirms the importance of continuous molecular surveillance for effective vaccine strategy formulation.

### 3.5. Temporal Trend of FMDV SAT 2 Lineages in Ethiopia (1957–2025)

FMDV serotype SAT 2 presents a dynamic and challenging history in Ethiopia, initially being reported in bovine populations in the southern Sidama and Borena areas between 1989 and 1991 [30]. Following a remarkable epidemiological gap spanning approximately 16 years, the serotype re‐emerged in 2007 [15] in areas such as Asosa, establishing a pattern of consistent detection in outbreaks that continues through 2023 [6, 15, 16]. This resurgence marked a shift toward wider distribution; for instance, by 2010, the virus was confirmed in central Ethiopia including Debre Zeit, Debre Birhan, Addis Ababa, and Sululta [33, 34, 42]. Subsequent years saw accelerated spread, with SAT 2‐associated outbreaks in Addis Ababa and East Wollega in 2014, leading to widespread detections in 2015 across East and North Shoa, Sidama, Arsi, and East Shewa (Adama and Boset) [16, 33, 46]. Furthermore, the virus demonstrated an expansion in its host range, affecting both bovine and swine species in locations like Gambella (Lare) and Debre Birhan [16] and infecting both cattle and small ruminants during the 2015 widespread outbreaks. More recently, the virus has been reported in northern areas, including Kafta Humera (2018), and confirmed in Aba’ala District (Afar Region) and Tigray (Southern Zone, Mekele) in 2019 [16, 49], underscoring its national persistence and increasing geographical reach.

Outbreaks persisted into 2020, with SAT2 detected in Horoguduru and Jimma Genet areas [6], followed by additional outbreaks in 2021 in the Jimma Zone and in Bishoftu, Modjo, Fitche, DebreBirhan, and Addis Ababa [52]. The virus continued to spread in 2022, affecting East, North, and South West Shoa, Afar, Jimma, and WolaitaSodo areas [6, 54]. In 2023, SAT2 was again confirmed in Arsi and Wolayta zones [6], while the AHI (unpublished) reported further outbreaks in West Arsi, Arsi, and Horoguduru Wollega during 2023 and 2024.

**Figure 5:**
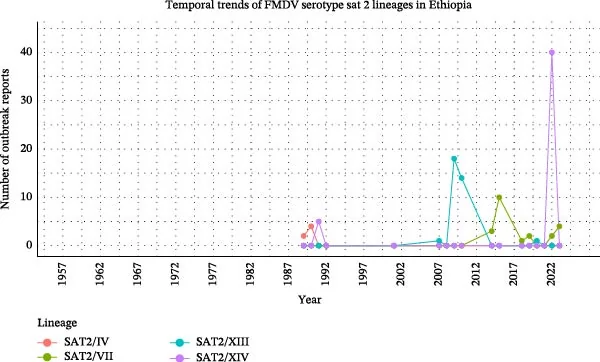
Temporal dynamics of foot‐and‐mouth disease virus SAT 2 lineages, 1957–2025.

The evidence concerning FMDV serotype SAT 2 reveals its status as a persistent and recurrent threat within the Ethiopian livestock sector, circulating across multiple regions for more than three decades, affecting diverse species, and demonstrating recurring waves of transmission across wide geographic areas. This pattern of initial presence, followed by a prolonged period of absence and subsequent re‐establishment highlights an intermittent yet deeply rooted endemic challenge. Further molecular analysis, utilizing phylogenetic sequencing of the VP1 gene, indicates substantial viral diversity, confirming the cocirculation of four distinct SAT 2 topotypes IV, VII, XIII, and XIV [6, 16]. This diversity strongly implies either multiple independent introductions into Ethiopia or significant localized evolution. Historically, the early detection (1991) was linked to the prevalent topotype XIV, whose lineage can be traced back to ancestors circulating earlier in the 20th century. Notably, the overall circulation pattern is characterized by a sequential emergence of different lineages, as detailed visually in Figure 5, where one particular lineage tends to achieve temporary dominance. This lineage turnover is a crucial finding as the presence of multiple, distinct topotypes, with one often replacing another as the dominant circulating strain, has significant implications for the continuous efficacy of surveillance programs and the appropriate selection of vaccine strains.

The recent epidemiological history of FMDV serotype SAT 2 in Ethiopia is defined by a dynamic turnover of viral lineages and a dramatic resurgence that has altered the national serotype profile.

Circulating FMDV topotypes have exhibited distinct temporal shifts. Topotype XIII was predominant during 2009–2010 and again in 2020, while various lineages of Topotype VII (Alx‐12 and Lib‐12) circulated between 2014 and 2018 [16, 58]. Most significantly, the period from 2019 to 2023 was marked by an increase in SAT 2 prevalence, establishing it as the most frequently reported serotype. Regarding the re‐emergence of Topotype XIII, interpretation remains nuanced; while phylogenetic data confirm the consistency of the topotype, it is unclear whether this reflects the persistence of an enzootic lineage or discrete, independent reintroduction events. Given the high degree of transboundary livestock mobility, both scenarios are plausible, although emerging genomic evidence suggests that intermittent reintroductions may be a primary driver in maintaining the observed topotype dominance.

This resurgence was fundamentally linked to the re‐emergence and expansion of the SAT2/XIV topotype, which was detected again in 2022–2023 after a long absence [6]. This shift is quantitatively evidenced by SAT 2, accounting for 49 detections compared to 27 for FMDV O and only 1 for FMDV A during 2019–2023 [6].

While this review focuses on Ethiopia, these results must be viewed within the context of the East African FMDV ecosystem (Pool 4). The serotype and topotype circulation patterns identified here align with regional observations across the Horn of Africa, confirming that Ethiopia is an integral part of the broader transboundary circulation. However, the distinct epidemiological clusters identified in this study also highlight the influence of Ethiopia’s unique agroecological zones and intensive livestock trade, which may drive transmission dynamics that differ from those in neighboring countries.

The sustained endemicity of FMDV in Ethiopia is driven by the rapid evolutionary turnover of viral lineages, which frequently leads to the emergence of novel topotypes and complicates long‐term vaccine matching strategies. Re‐established circulation was initially observed in the western (Gambela and Benishangul‐Gumuz) and central (Oromia) regions of Ethiopia in 2009, with subsequent southward expansion by 2015 [16]. This expanding range is primarily driven by livestock movements, which facilitate internal dissemination and underpin the cross‐border introduction of the re‐emerging FMDV SAT‐2 Topotype XIV into Western Asian countries, including Turkey, Iraq, and Oman [35].

These transcontinental dissemination patterns are heavily influenced by livestock trade networks connecting the Horn of Africa with the Arabian Peninsula. Ethiopia, a major exporter of live animals, utilizes both formal and informal trade routes, specifically those originating from pastoral areas such as Borena to supply Middle Eastern markets. Movement along these traditional corridors, often characterized by limited digital traceability and insufficient sanitary oversight, creates high‐risk pathways for the rapid export of the virus [62, 63]. Given that SAT‐2 has been largely absent from West Asia and Europe for decades, the resident livestock populations are immunologically naïve; this contrasts with established regional immunity to serotypes O and A, allowing the Ethiopian SAT‐2/XIV lineage to spread with explosive speed upon entry.

Furthermore, the internal distribution of these topotypes is significantly shaped by ecosystem diversity and the adaptation of pastoral practices to seasonal climate and grazing trends [62, 64]. While these movements are essential for trade and pastoral livelihoods, they facilitate contact between domestic cattle and susceptible wildlife, further complicating disease control. Although these established pathways likely contribute to regional dissemination, the precise transmission dynamics and directionality remain difficult to confirm in the absence of comprehensive longitudinal genomic tracing and real‐time animal movement data [35].

### 3.6. Spatial Distribution of FMDV Serotypes in Ethiopia (1957–2025)

**Figure 6:**
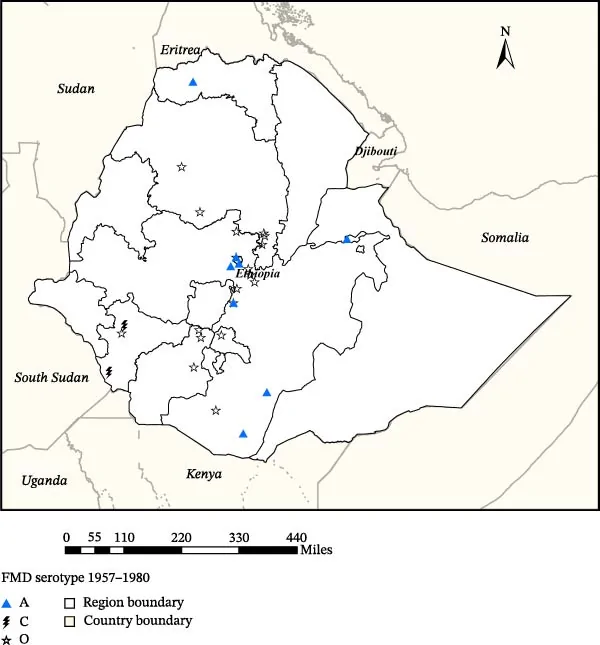
Spatial distribution of FMDV serotypes in Ethiopia (1957–1980).

**Figure 7:**
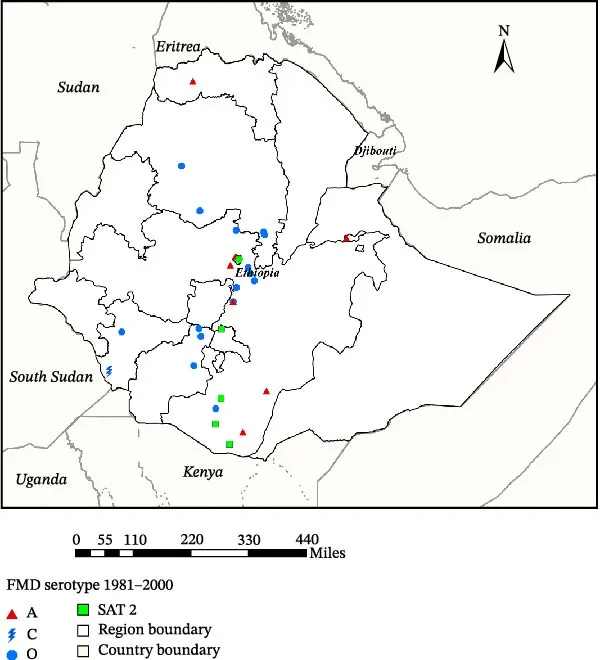
Spatial distribution of FMDV serotypes in Ethiopia (1981–2000).

**Figure 8:**
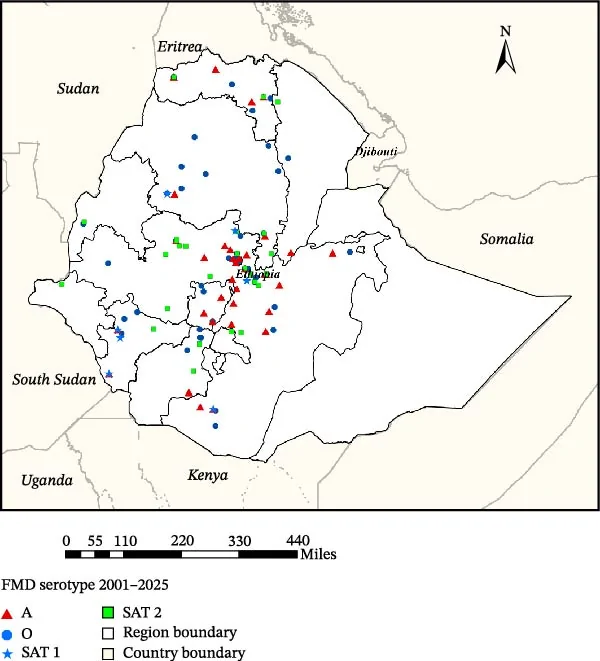
Spatial distribution of FMDV serotypes in Ethiopia (2001–2025).

The geographic distribution of FMDV serotypes in Ethiopia shows a clear shift over time. During Period I (1957–1979), recorded outbreaks were few and concentrated primarily in central and southwestern Ethiopia (Figure 6). However, this distribution likely reflects spatial bias in surveillance coverage and accessibility rather than the true epidemiological range of the virus during that period. Three serotypes, O, A, and C, were observed, where serotype C appeared mostly in the southwest, while A and O were concentrated in central Ethiopia, leaving much of the country unrepresented (Figure 7). In Period II (1980–2000), the distribution pattern changed: serotype C appeared once again in the southwest and then disappeared, while SAT 2 emerged for the first time, spreading through southern, western, and central Ethiopia (Figure 7). Serotype O expanded widely, covering almost the entire country except the Afar and Somali lowlands. Outbreak investigations remained largely focused around central Ethiopia, likely due to accessibility and livestock movement toward Addis Ababa (Figure 8). In Period III (2001–2025), both the number and spatial coverage of outbreak reports increased significantly, extending into northern regions, including Amhara and Tigray. SAT 1 appeared for the first time, SAT 2 became more established, and serotypes A and O continued to persist, with O maintaining dominance (Figure 8). Overall, there is progressive geographic expansion of outbreak reporting and a shifting serotype presence across Ethiopia over time.

To contextualize these findings within a broader epidemiological framework, the FMDV landscape globally encompasses seven distinct serotypes partitioned across seven regional virus pools. Among these, East Africa (Virus Pool 4) represents one of the most dynamic and complex disease settings worldwide, hosting multiple endemic serotypes—specifically O, A, SAT 1, SAT 2, and SAT 3 [16, 62].

While only a subset of these serotypes cocirculates simultaneously in other global pools (such as Pool 1 in Southeast Asia or Pool 2 in South Asia, where entirely different topotypic constellations predominate), Ethiopia acts as a focal point of extraordinary genetic diversity. Within Ethiopia and the immediate Horn of Africa region, viral persistence is driven by multiple active topotypes and lineages: notably, the East Africa‐3 (EA‐3) and EA‐4 topotypes for serotype O, dynamic lineage turnover within the Africa topotype (such as genotypes G‐I, G‐IV, and G‐VII) for serotype A, and topotypes IV, XIV, and XIII for serotype SAT 2 [6, 35, 62].

This high concentration of multiple evolving topotypes and continuous lineage cocirculation within a single epidemiological corridor highlight the unique selective pressures operating in the region. Consequently, this local spectrum captures a substantial portion of the broader continental diversity observed across sub‐Saharan Africa, emphasizing why routine, localized strain characterization and continuous vaccine‐matching surveillance are indispensable components for mitigating the impact of transboundary incursions.

### 3.7. Vaccine Studies of FMDV in Ethiopia

The national FMD control strategy in Ethiopia is critically compromised by a profound deficit in vaccine availability and efficacy. Local production capacity at the NVI is demonstrably insufficient, providing fewer than 500,000 doses annually against an estimated national requirement that surpasses three million doses. Candidate vaccine strains demonstrated generally high antigenic similarity with most serotype O and SAT2 field isolates; however, a single serotype A isolate exhibited poor antigenic matching, yielding r1 values of 0.14 and 0.25 that fell below the accepted threshold for vaccine compatibility [65].

More recently, vaccine‐matching assessments showed that the vaccine strain O/ETH/38/2005 was antigenically matched to only 10 of 16 serotype O field isolates, with poorer matching observed among some EA‐4 prototype viruses, a shortfall that is particularly impactful within crucial delineated export sourcing areas of southern Ethiopia [6, 60].

Beyond the quantitative shortage, the absence of timely, systematic serological and molecular characterization of field strains presents a significant qualitative challenge: it obstructs the essential process of vaccine matching, making it difficult to guarantee that the locally produced vaccine strains are antigenically relevant to the constantly evolving and circulating FMD viruses. While a limited number of studies have touched upon vaccine matching in Ethiopia, comprehensive and systematic investigations into the antigenic relationship between current vaccines and field isolates remain critically limited, highlighting a significant knowledge gap that necessitates a rigorous review of current practices and available data.

Antigenic matching studies provide evidence that vaccine field strain compatibility varies among the circulating Ethiopian FMDV strains. A two‐dimensional virus neutralization study demonstrated a significant antigenic variation and distinct mismatch profiles among lineages, highlighting the need for continuous vaccine‐matching surveillance and periodic vaccine strain evaluation [47].

## 4. Limitations of This Review

The scope of this comprehensive review, which analyzes FMDV circulation in Ethiopia over the period 1957–2025, is subject to inherent limitations stemming from variability in reporting, data accessibility, and the evolving diagnostic methodologies used across nearly seven decades.

First, the complete retrieval of all relevant documentation was partially restricted due to limited access to certain premium‐subscription databases. Despite this constraint, rigorous efforts were undertaken to ensure that all highly relevant, publicly accessible, and peer‐reviewed papers available through comprehensive search engines and institutional repositories were thoroughly evaluated and integrated. While we recognize that advanced spatial and temporal modeling would further enrich the analysis, the heterogeneous nature of this historical dataset reflecting shifts from passive reporting in the early periods to more active, targeted surveillance in recent years limits the suitability of such complex statistical approaches. Consequently, we have focused this work on the systematic curation and baseline characterization of these records. We believe that this comprehensive database serves as a foundational resource, providing the essential evidence base required for future predictive modeling, phylogeographic studies, and regional comparative analyses.

Second, and perhaps the most significant methodological limitation for historical assessment, the criteria for inclusion mandated a focus on FMD serotypes confirmed exclusively by high‐certainty diagnostics, specifically antigen detection ELISA and molecular methods, such as phylogenetic analysis. This necessary criterion resulted in the systematic exclusion of serotypes identified solely through serological assays (e.g., solid‐phase ELISA). This is a salient point as the bulk of historical FMD research in Ethiopia has focused primarily on generating serological prevalence data, meaning this review potentially underrepresents the full historical spectrum of FMD virus circulating serotypes for which definitive molecular confirmation is currently unavailable.

A primary constraint of this review is the systemic deficiency in national FMD surveillance, characterized by frequent underreporting and limited investigation of outbreaks. This lack of robust documentation is further compounded by significant spatial and temporal biases across regions and timeframes, suggesting that the synthesized data substantially underestimate the true epidemiological burden and viral circulation dynamics in Ethiopia. Furthermore, the findings are subject to an inherent publication bias; as studies documenting confirmed outbreaks or positive viral detections are more frequently published than those reporting null or negative results, the observed prevalence and geographic distribution of FMDV serotypes may reflect a selection bias. These limitations must be considered when interpreting the overall epidemiological landscape.

## 5. Conclusion and Recommendation

Effective FMD control in Ethiopia faces persistent challenges, necessitating a comprehensive, multifaceted strategy that integrates vaccination, strict movement controls, and robust community engagement. This review specifically analyzed the FMD epidemiological landscape during three periods (Period I–III) from 1957 to 2025. The findings highlighted the continued significance of serotypes O, A, and SAT 2. The genotypes within each serotype exhibit a distinct divergence, pointing to an active epidemiological shift. Serotype O dominated the FMD virus distribution in the country, primarily represented by the EA‐3 and EA‐4 lineages. Serotype A recently demonstrated the endemic establishment of the A/Africa G‐IV lineage. The most critical observation is the significant and increasing dominance of FMDV SAT 2 topotype XIV. This trend strongly suggests a critical shift in the circulating SAT 2 strains within Ethiopia, demanding immediate attention for national control programs. Serotype C has not been reported since 1983, indicating a likely extinction from the Ethiopian FMD landscape. Serotype SAT 1 though molecularly characterized in 2007, subsequent low‐level detection of SAT 1 has been exclusively through serological methods, either antigen detection ELISA or solid‐phase competitive ELISA. The lack of recent molecular confirmation for SAT 1 raises significant question about its current existence as a causative agent of FMD outbreaks in Ethiopia and highlights a critical deficiency in confirmatory diagnostics and reporting.

Based on the evidence of evolving FMDV genetics and strain dominance identified in this comprehensive review, the following actions are essential for enhancing FMD control and prevention in Ethiopia:

1. Prioritize integrated surveillance and molecular characterization: Establish and implement a robust, integrated surveillance system that actively involves livestock owners to significantly improve monitoring, reporting, and rapid outbreak investigation and response.
2. Continuous molecular characterization (genotyping) of circulating FMDV strains is vital to accurately track the observed divergence and confirm the emergence of new FMDV lineages, including SAT 2 topotype XIV, O EA‐4, and A/Africa G‐IV.
3. Confirm SAT 1 status: Specifically, all future suspected outbreaks involving SAT 1 must be subjected to immediate and comprehensive molecular analysis to definitively confirm its circulation and resolve the current ambiguity surrounding its true epidemiological status.
4. The strengthening of diagnostic infrastructure to achieve a national mean laboratory confirmation time of <7 days, while simultaneously utilizing standardized performance indicators and systematic pipeline monitoring to evaluate the surveillance system and ensure timely detection and response.
5. There is an urgent need to review the current FMD vaccine composition. The vaccines must be verified to ensure that they will provide effective cross‐protection against the emerging and dominant FMDV lineages, specifically SAT 2 topotype XIV, serotype O EA‐4, and A/Africa G‐IV, to maintain vaccine efficacy in the field.
6. Enforce and significantly improve compliance with existing livestock movement restrictions to curb the rapid dissemination of highly mobile strains. This must be coupled with efforts to boost farm‐level biosecurity measures through targeted community engagement, public awareness campaigns, and practical training programs.

## Author Contributions

**Daniel Gizaw, Fentahun Wondmnew, and Fasil Aklilu:** conceptualization, methodology, investigation, formal analysis, writing – original draft. **Getnet Abie Mokonnen:** investigation, visualization, writing – review and editing. **Nina Henning**: funding acquisition, project administration, writing – review and editing. **Berhanu Admasu**: resources, supervision, validation, writing – review and editing. **Badi Maulidi**: methodology, validation, writing – review and editing. **Wudu Temesgen**: conceptualization, supervision, formal analysis, writing – review and editing. **Jef. Hammond**: conceptualization, supervision, writing – review and editing.

## Funding

No funding was received for this manuscript.

## Ethics Statement

This review relied exclusively on secondary, publicly available, or institutional data; therefore, ethical approval was not required. Institutional data obtained under permission were handled confidentially, and all sources were properly acknowledged to ensure research integrity and transparency.

## Conflicts of Interest

The authors declare no conflicts of interest.

## Data Availability

The datasets generated and/or analyzed during the current study are available within the manuscript.
